# Supplementary material for: Plasmodium falciparum sexual parasites regulate infected erythrocyte permeability
Source: Commun Biol. 2020 Dec 1;3:726. doi: 10.1038/s42003-020-01454-7 (PMC7708629; doi:10.1038/s42003-020-01454-7)
Supplement: Supplementary file 1 — Supplemental Information [file 42003_2020_1454_MOESM1_ESM.pdf]

Plasmodium falciparum **sexual parasites regulate infected erythrocyte permeability**

Guillaume Bouyer*[1,2], Daniela Barbieri*[2,3], Florian Dupuy[2,3], Anthony Marteau[2,3], Abdoulaye Sissoko[2,4], Marie-Esther N'Dri[2,3], Gaelle Neveu[2,3], Laurianne Bedault[2,3], Nabiha Khodabux[2,3], Diana Roman[2,4], Sandrine Houzé[2,4], Giulia Siciliano[5], Pietro Alano[5], Rafael M. Martins[6], Jose-Juan Lopez-Rubio[6], Jérome Clain[2,4], Romain Duval[2,4], Stéphane Egée #[1,2], Catherine Lavazec #[2,3]

SUPPLEMENTARY INFORMATION

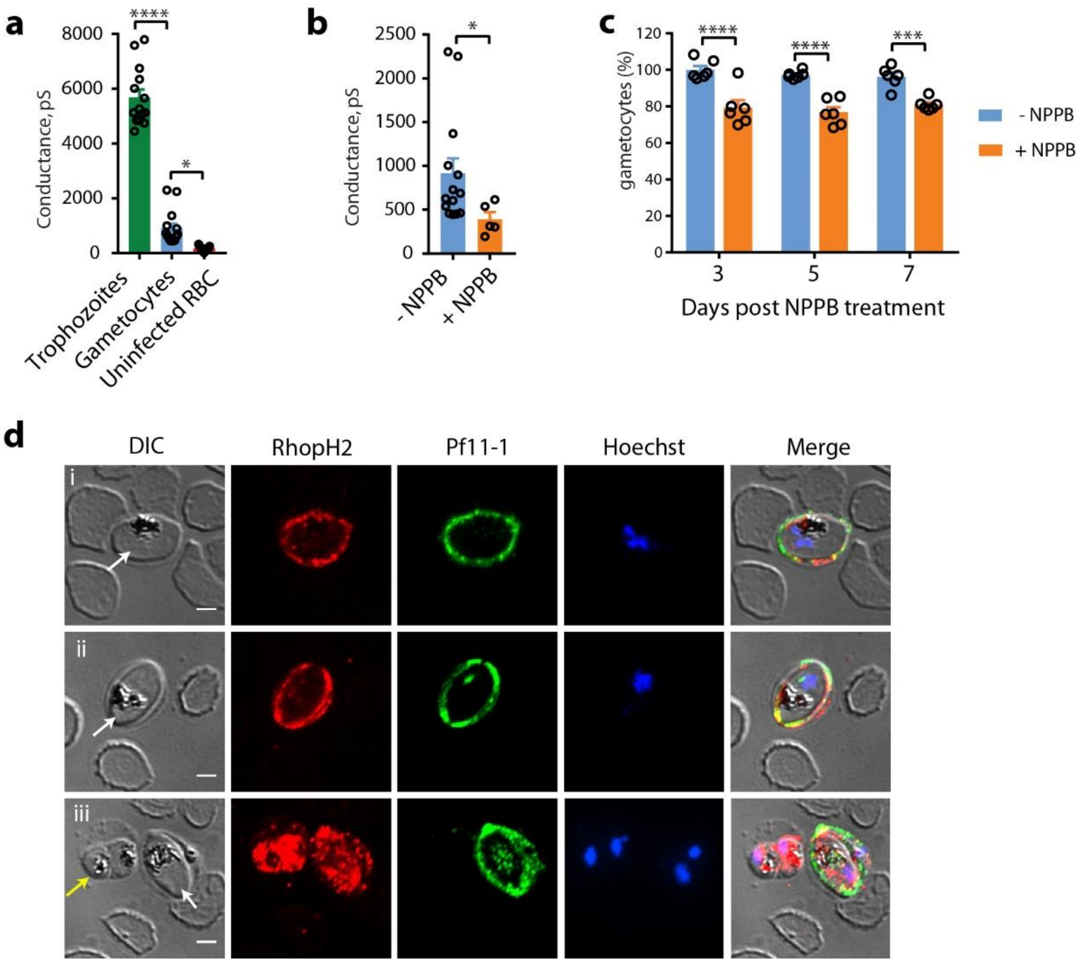

Supplementary Figure S1

a. Whole cell conductance calculated at -100 mV determined by patch clamp experiments on erythrocytes infected with trophozoites, stage II gametocytes and uninfected erythrocytes. **b.** Whole cell conductance calculated at -100 mV determined by patch clamp experiments on stage II GIE in

presence or absence of NPPB. **c.** Gametocytemia at 3 days, 5 days or 7 days of culture after a 3-hour incubation of stage II GIE with or without 100 µM NPPB followed by two washes in RPMI. The graph is normalized by the gametocytemia of control (without NPPB) at 3 days post treatment. In a-c, circles indicate the number of independent experiments. Error bars show the standard error of the mean (SEM). **d.** Immunofluorescence analysis of acetone/methanol-fixed infected erythrocytes stained with mouse antibodies against the NPP component RhopH2 (red) and rabbit antibodies against the gametocyte-specific protein Pf11-1 (green), showing the presence of RhopH2 in immature GIE. Yellow arrow: asexual stages, white arrows: immature GIE. DNA is stained with Hoechst 33342 (blue). Bars represents 5 µm.

a

	stage I	stage II	stage III	stage IV	stage V
average gametocytemia (%)	2.10	1.58	1.39	0.78	1.64
range (%)	1.19 - 3.45	0.55 - 4.31	0.58 - 2.07	0.5 - 1.3	0.68 - 3.18

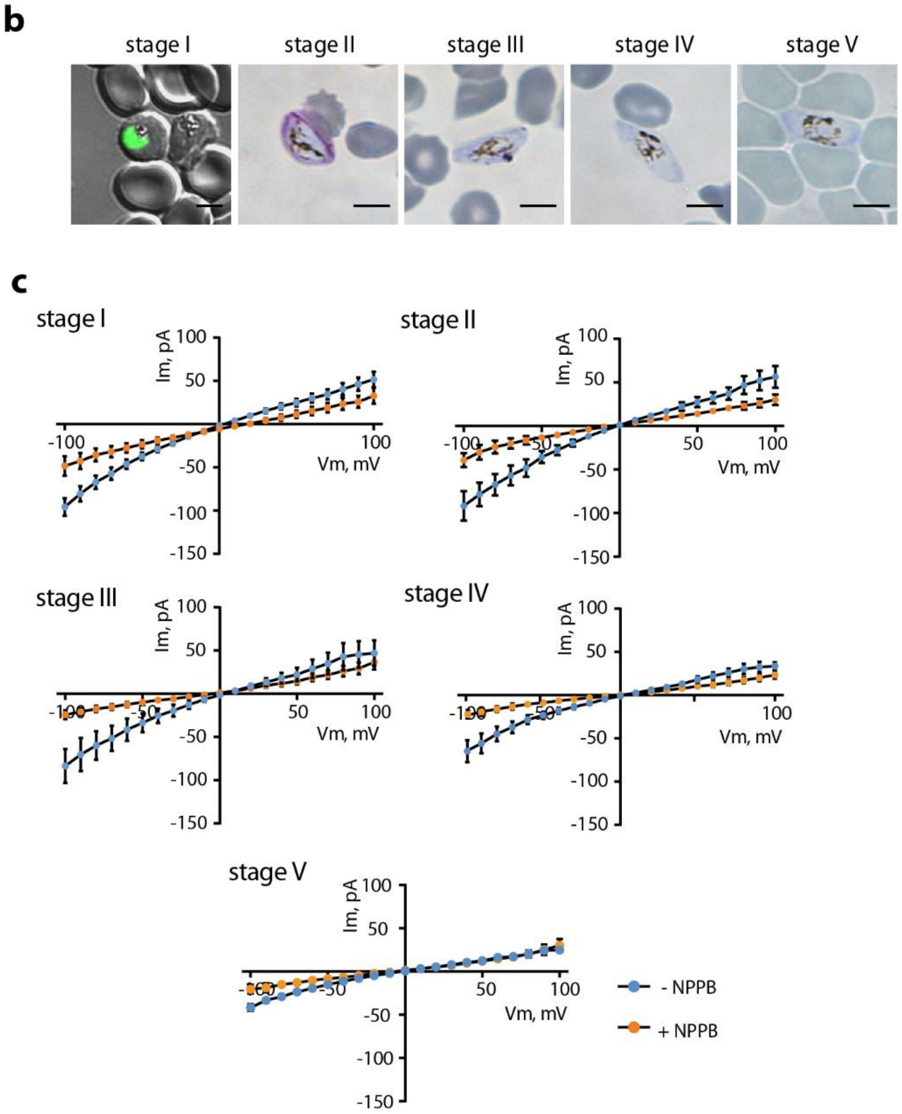

Supplementary Figure S2

a. Average gametocytemia in NF54 cultures used for isosmotic lysis experiments. **b.** Representative pictures of stage I GIE from the NF54-pfs47-pfs16-GFP line as observed by fluorescence microscopy and of stage II, III, IV and V GIE from NF54 as observed in Giemsa-stained thin blood smears. Scale bars: 5 µm. **c.** I-V plots from patch experiments on GIE from stage I to stage V with (orange) or without (blue) 100 µM NPPB.

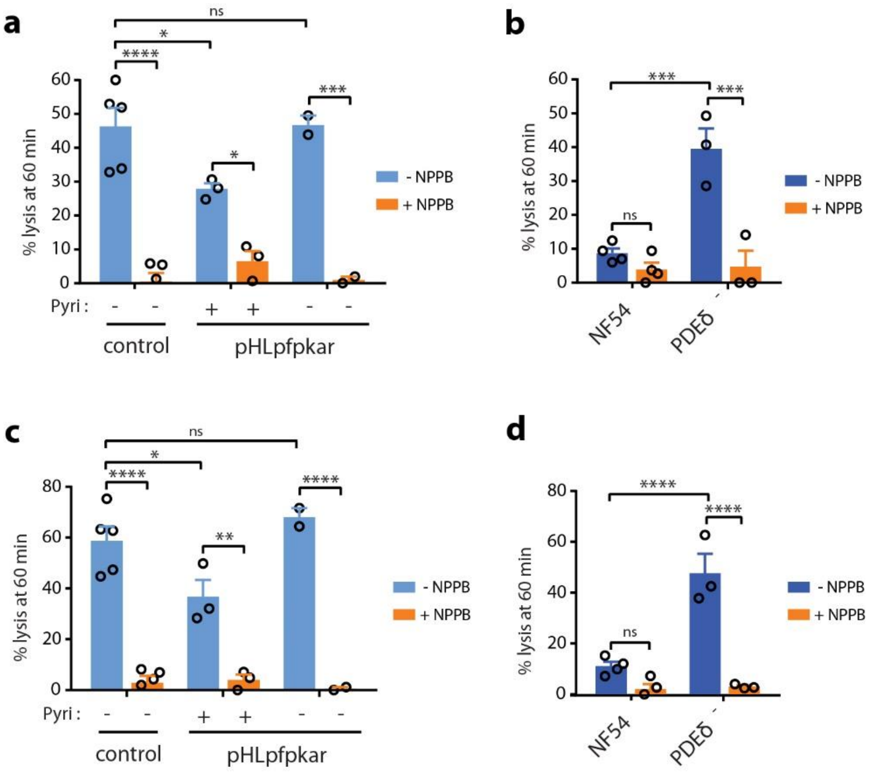

Supplementary Figure S3

a, c. Alanine-induced (a) and PhTMA^{+}-induced (c) isosmotic lysis of stage II GIE from the NF54 isolate (Control) and the transgenic pHL*pfpkar* line, cultivated with and without pyrimethamine (Pyri).

b, d. Alanine-induced (b) and PhTMA^{+}-induced (d) isosmotic lysis of stage V GIE from the NF54 isolate and the transgenic line PDEδ^{-}. All experiments were performed in presence or absence of 100 µM NPPB. Circles indicate the number of independent experiments, error bars show the SEM and statistical significance is determined by one-way ANOVA with Sidak correction for multiple comparisons.

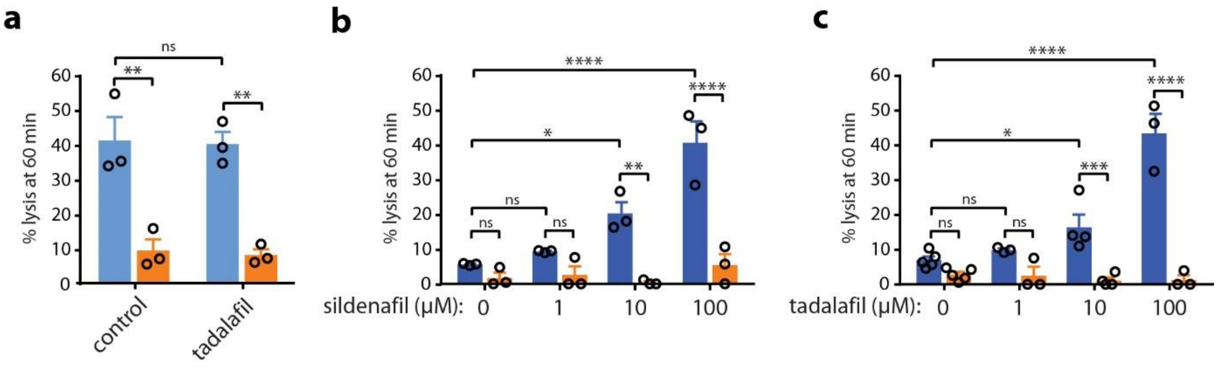

Supplementary Figure S4.

a. Sorbitol-induced isosmotic lysis of stage II GIE with 100 µM tadalafil. **b, c.** Sorbitol-induced isosmotic lysis of stage V GIE with 1 µM, 10 µM or 100 µM sildenafil (b) or with 1 µM, 10 µM or 100 µM tadalafil (c). All experiments were performed in presence (orange) or absence (blue) of 100 µM NPPB. Circles indicate the number of independent experiments, error bars show the SEM and statistical significance is determined by one-way ANOVA with Sidak correction for multiple comparisons.

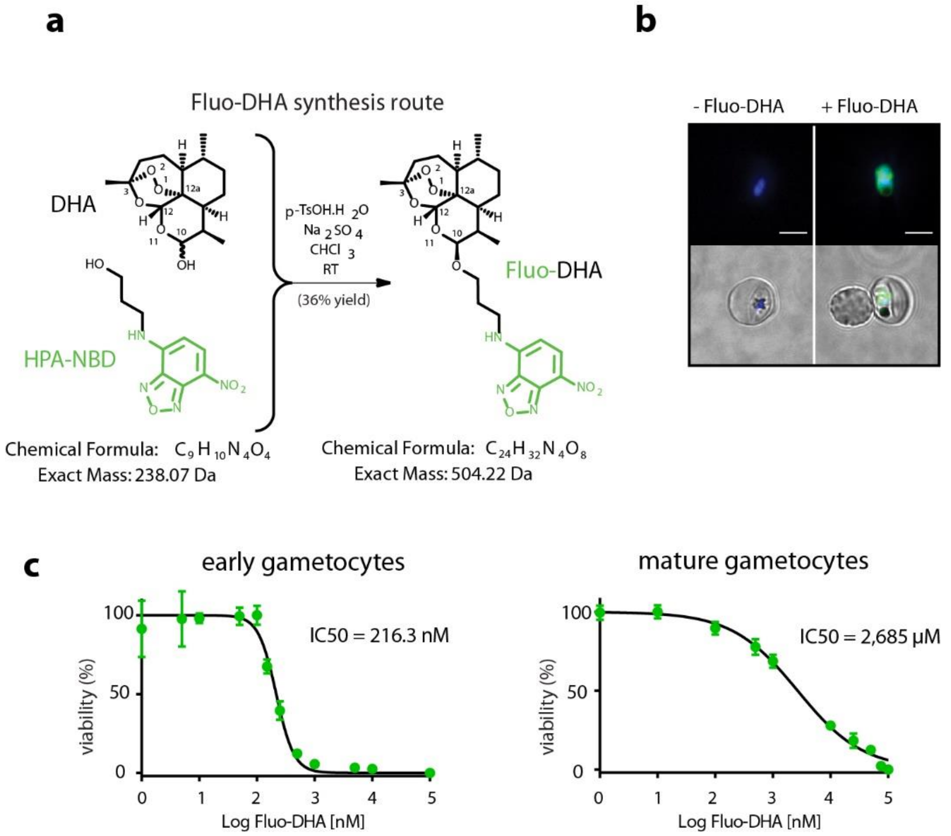

Supplementary Figure S5.

a. Schematic of Fluo-DHA synthesis from dihydroartemisinin (DHA) and the fluophore HPA-NBD. **b.** Fluorescence microscopy imaging of paraformaldehyde-fixed early GIE showing Fluo-DHA uptake (green). DNA is stained with Hoechst 33342 (blue). Scale bars: 5 µm. **c.** Dose-response curves and IC_{50} values for Fluo-DHA in early gametocytes (left panel) and mature gametocytes (right panel) from the NF54-cg6-pfs16-CBG99 line. Viability (luciferase activity) was determined 72 hours after a 3-hour incubation with serial dilutions of Fluo-DHA. Error bars show the SEM.

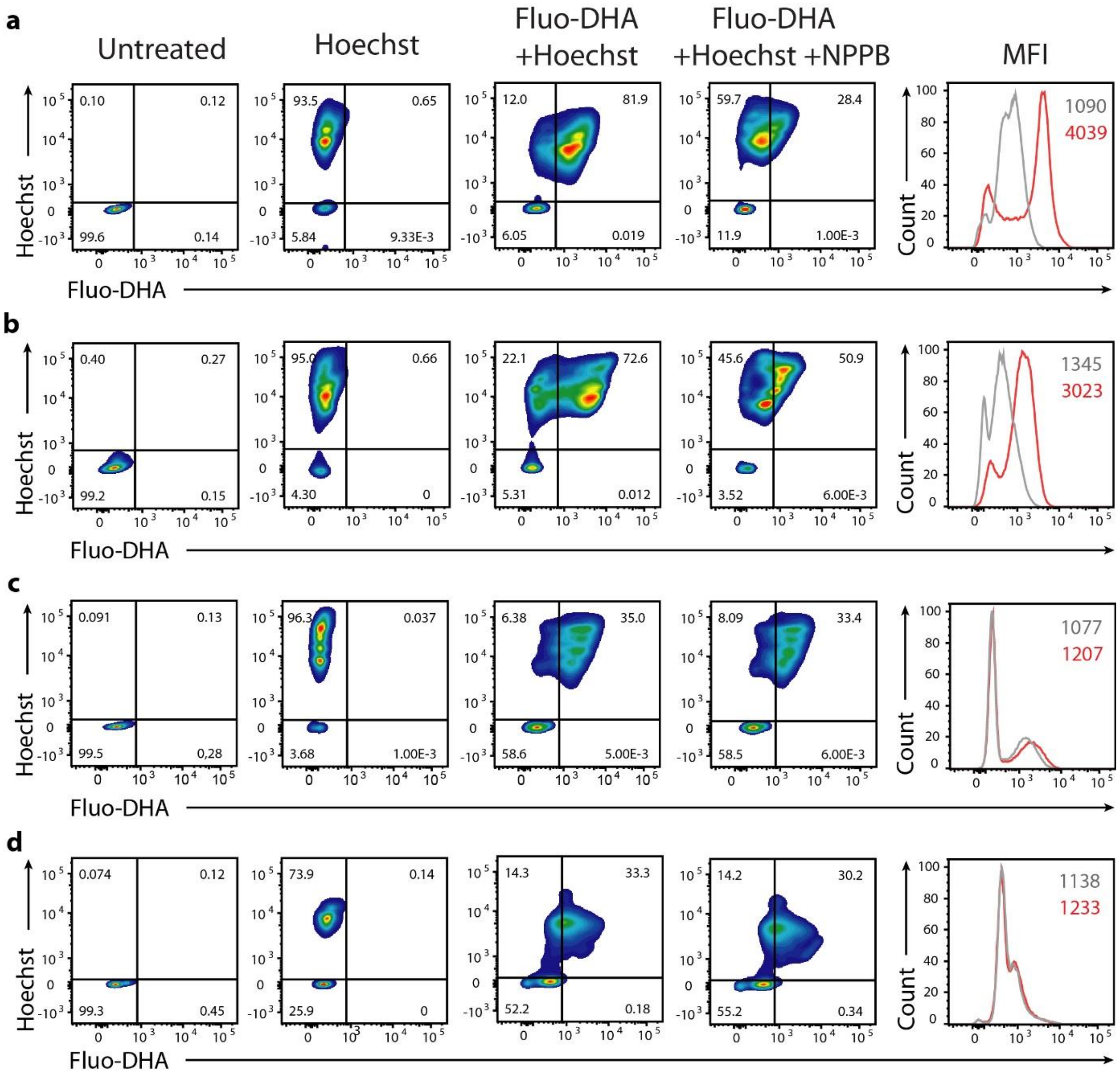

Supplementary Figure S6.

Scatter plots showing the gating strategy for Fluo-DHA uptake. GIE from stage II (a), III (b), IV (c) or V (d) were pre-incubated or not with 100 µM NPPB for 30 minutes and then incubated with 1 µM of Fluo-DHA for 2 hours. Twenty minutes before the end of incubation, GIE were stained with Hoechst 33342. Values in black: percentages of cells, values in grey and red: mean of fluorescence intensities (MFI) of Fluo-DHA with NPPB (grey) or without NPPB (red). Representative panels for each stage are shown.

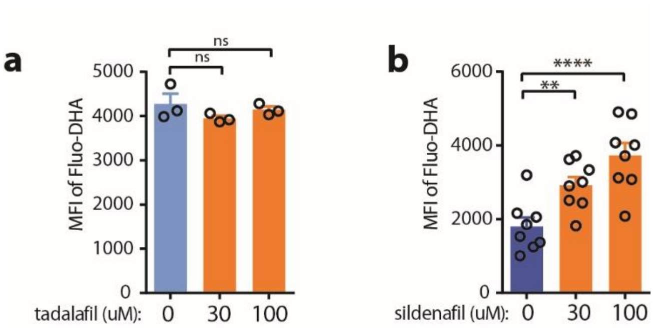

Supplementary Figure S7.

a. Fluo-DHA uptake in stage II GIE upon 30 µM or 100 µM tadalafil incubation. **b.** Fluo-DHA uptake in stage V GIE upon 30 µM or 100 µM sildenafil incubation. Circles indicate the number of independent experiments, error bars show the SEM and statistical significance is determined by one-way ANOVA with Dunnett correction for multiple comparisons.
